# Supplementary material for: Molecular analysis of acute pyelonephritis—excessive innate and attenuated adaptive immunity
Source: Life Sci Alliance. 2024 Dec 20;8(3):e202402926. doi: 10.26508/lsa.202402926 (PMC11662066; doi:10.26508/lsa.202402926)
Supplement: Supplementary file 1 [file LSA-2024-02926_TableS1.docx]

**Table S1**. Patient characteristics, laboratory samples and urine culture, Cohort I.

| **Patient characteristics (N = 111)** |  | **1^st^ DMSA+ association** | **2^nd^ DMSA+ association** |
| --- | --- | --- | --- |
| **Age (months)** |  | *P* = 0.21 | *P* = 0.45 |
| Mean (range) | 13.2 (1-156) |  |  |
| Median (mode) | 5 (5) |  |  |
| ≤12 months, no. (%) | 93 (83.8) |  |  |
| 13-24 months, no. (%) | 7 (6.3) |  |  |
| >24 months, no. (%) | 11 (9.9) |  |  |
| **Gender, no. (%)** |  | *P* = 0.01 | *P* = 0.64 |
| Male | 61 (55) |  |  |
| Female | 50 (45) |  |  |
| Male:Female | 1.2:1 |  |  |
| **White blood cell count (x10^9^/L) (N = 111)** | | | |
| Mean±SD | 18.86±8.21 |  |  |
| Median | 17.99 |  |  |
| <15 x10^9^/L, no. (%) | 37 (35.5) |  |  |
| >15 x10^9^/L, no. (%) | 74 (64.5) | *P* = 0.31 | *P* = 0.24 |
| **CRP (mg/L) (N = 108)*** |  |  |  |
| ≥50 mg/L**, no. (%) | 78 (72) | *P* = 0.04 | *P* = 0.09 |
| Mean±SD | 100.6±72.3 |  |  |
| Median (range) | 92.3 (1.4-375.3) |  |  |
| ≤10 mg/L, no. (%) | 5 (4.6) |  |  |
| >10 mg/L, no. (%) | 103 (95.4) |  |  |
| **Uropathogen, no. (%) (N = 110)***** |  | *P* = 0.32 | *P* = 0.11 |
| *Escherichia coli* | 97 (88.2) |  |  |
| Non *Escherichia coli* | 13 (11.8) |  |  |
| *Klebsiella* | 5 |  |  |
| *Pseudomonas* | 2 |  |  |
| *Enterobacter* | 2 |  |  |
| *Citrobacter* | 1 |  |  |
| *Proteus* | 1 |  |  |
| Group B *Streptococcus* | 1 |  |  |
| *Enterococci* | 1 |  |  |
| CRP = C-reactive protein.  *P*<0.05 is considered as significant.  * CRP not available in 3 patients.  ** CRP≥50 mg/L is considered as significant increase in CRP.  *** Negative urine culture in 1 patient with 1^st^ DMSA positive. | | | |
