# Supplementary material for: Molecular analysis of acute pyelonephritis—excessive innate and attenuated adaptive immunity
Source: Life Sci Alliance. 2024 Dec 20;8(3):e202402926. doi: 10.26508/lsa.202402926 (PMC11662066; doi:10.26508/lsa.202402926)
Supplement: Supplementary file 2 [file LSA-2024-02926_TableS2.docx]

**Table S2**. Patient Characteristics, laboratory samples and urine culture, Cohort II.

| **Patient characteristics (N = 52)** |  | **1^st^ DMSA+ association** | **2^nd^ DMSA+ association** |
| --- | --- | --- | --- |
| **Age (days)** |  | *P* = 0.86 | *P* = 0.36 |
| Mean (range) | 127.4 (7-349) |  |  |
| Median (95% CI) | 104.5 (67-144) |  |  |
| ≤ 3 months, no. (%) | 24 (46) |  |  |
| 3-6 months, no. (%) | 14 (27) |  |  |
| >6 months, no. (%) | 14 (27) |  |  |
| **Gender, no. (%)** |  | *P >* 0.99 | *P* = 0.06 |
| Male | 22 (42) |  |  |
| Female | 30 (58) |  |  |
| Male:Female | 1:1.4 |  |  |
| **CRP (mg/L)** |  |  |  |
| ≥50 mg/L**, no. (%) | 43 (83) | *P* = 0.11 | *P* = 0.45 |
| Mean±SD | 95.92±50.9 |  |  |
| Median (range) | 97.5 (3-247) |  |  |
| ≤10 mg/L, no. (%) | 3 (5.8) |  |  |
| >10 mg/L, no. (%) | 49 (94.2) |  |  |
| **Uropathogen, no. (%)** |  | *P >* 0.99 | *P* = 0.07 |
| *Escherichia coli* | 48 (92.3) |  |  |
| Non *Escherichia coli* | 4 (7.7) |  |  |
| *Klebsiella* | 3 |  |  |
| *Enterococci* | 1 |  |  |
| CRP = C-reactive protein.  P<0.05 is considered as significant.  ** CRP≥50 mg/L is considered as significant increase in CRP. | | | |
