# Supplementary material for: Molecular analysis of acute pyelonephritis—excessive innate and attenuated adaptive immunity
Source: Life Sci Alliance. 2024 Dec 20;8(3):e202402926. doi: 10.26508/lsa.202402926 (PMC11662066; doi:10.26508/lsa.202402926)
Supplement: Supplementary file 3 [file LSA-2024-02926_TableS3.docx]

**Table S3**. Top regulated genes in acute compared to follow-up samples (adj. *P* < 0.05, FC > 1.5) FC = Fold Change.

| **Symbol** | **Entrez Gene Name** | **FC**  **Cohort I** | **FC**  **Cohort II** | **Type(s)** |
| --- | --- | --- | --- | --- |
| *CD177* | CD177 molecule | 45.1 | 47.8 | other |
| *MCEMP1* | mast cell expressed membrane protein 1 | 11.4 | 11.2 | other |
| *HP* | haptoglobin | 7.6 | 11.7 | peptidase |
| *ANXA3* | annexin A3 | 7.5 | 7.2 | enzyme |
| *MGAM2* | maltase-glucoamylase 2 (putative) | 7.1 | 7.8 | other |
| *CASP5* | caspase 5 | 7.0 | 9.7 | peptidase |
| *FCGR1A* | Fc gamma receptor Ia | 6.9 | 7.7 | transmembrane receptor |
| *ANKRD22* | ankyrin repeat domain 22 | 6.2 | 9.8 | transcription regulator |
| *VNN1* | vanin 1 | 6.1 | 5.3 | enzyme |
| *GPR84* | G protein-coupled receptor 84 | 5.7 | 9.6 | G-protein coupled receptor |
| *FCGR1BP* | Fc gamma receptor Ib, pseudogene | 5.6 | 6.6 | transmembrane receptor |
| *BASP1-AS1* | BASP1 antisense RNA 1 | 5.6 | 5.8 | other |
| *FAM20A* | golgi associated secretory pathway pseudokinase | 5.5 | 7.0 | other |
| *LINC02967* | long intergenic non-protein coding RNA 2967 | 5.4 | 4.8 | other |
| *ALPL* | alkaline phosphatase, biomineralization associated | 5.4 | 6.0 | phosphatase |
| *MMP9* | matrix metallopeptidase 9 | 5.3 | 3.8 | peptidase |
| *S100A12* | S100 calcium binding protein A12 | 5.2 | 4.9 | other |
| *SOCS3* | suppressor of cytokine signaling 3 | 4.4 | 5.7 | phosphatase |
| *MMP8* | matrix metallopeptidase 8 | 4.0 | 8.1 | peptidase |
| *BMX* | BMX non-receptor tyrosine kinase | 3.8 | 5.8 | kinase |
| *OLFM4* | olfactomedin 4 | 2.0 | 5.6 | other |
| *FAM153A/B* | family with sequence similarity 153 member B | -1.6 | -3.7 | other |
| *LOC105376548* -- | | -1.8 | -2.9 | other |
| *SNORD3C* | small nucleolar RNA, C/D box 3C | -1.8 | -3.5 | other |
| *CCN3* | cellular communication network factor 3 | -1.8 | -3.1 | growth factor |
| *TNFRSF17* | TNF receptor superfamily member 17 | -2.0 | -3.2 | transmembrane receptor |
| *IGHG1* | immunoglobulin heavy constant gamma 1 (G1m) | -2.1 | -2.9 | other |
| *RHOXF1P1* | Rhox homeobox family member 1 pseudogene 1 | -2.2 | -3.0 | other |
| *UGT2B11* | UDP glucuronosyltransferase family 2 member B11 | -2.2 | -8.7 | enzyme |
| *CHI3L1* | chitinase 3 like 1 | -2.2 |  | enzyme |
| *FCRL6* | Fc receptor like 6 | -2.3 | -1.6 | other |
| *ADGRE4P* | adhesion G protein-coupled receptor E4 | -2.4 | -3.2 | other |
| *FGFBP2* | fibroblast growth factor binding protein 2 | -2.4 | -1.7 | other |
| *GNLY* | granulysin | -2.4 | -1.9 | other |
| *IL5RA* | interleukin 5 receptor subunit alpha | -2.5 | -2.8 | transmembrane receptor |
| *IGHG4* | immunoglobulin heavy constant gamma 4 (G4m) | -2.5 | -3.3 | other |
| *CLC* | Charcot-Leyden crystal galectin | -2.6 | -4.1 | enzyme |
| *RNF182* | ring finger protein 182 | -2.6 |  | enzyme |
| *SNORA14B* | small nucleolar RNA, H/ACA box 14B | -2.7 |  | other |
| *SIGLEC8* | sialic acid binding Ig like lectin 8 | -2.8 | -4.5 | transmembrane receptor |
| *IGHG3* | immunoglobulin heavy constant gamma 3 (G3m) | -2.9 | -3.4 | other |
| *LOC105370259* -- | | -3.0 | -5.1 | other |
| *TRGC2* | T cell receptor gamma constant 2 | -3.0 | -2.2 | other |
| *LOC105377267* -- | | -3.1 | -4.4 | other |
| *SNORA60* | small nucleolar RNA, H/ACA box 60 | -3.2 |  | other |
| *ALOX15* | arachidonate 15-lipoxygenase | -3.3 | -6.6 | enzyme |
