# Supplementary material for: Molecular analysis of acute pyelonephritis—excessive innate and attenuated adaptive immunity
Source: Life Sci Alliance. 2024 Dec 20;8(3):e202402926. doi: 10.26508/lsa.202402926 (PMC11662066; doi:10.26508/lsa.202402926)
Supplement: Supplementary file 4 [file LSA-2024-02926_TableS4.docx]

**Table S4.** Differentially expressed genes, 1^st^ DMSA+ vs. DMSA– (adj. *P <* 0.05 and log_2_ FC>1).

| **Symbol** | **Entrez Gene Name** | **Log_2_ FC** | **Adj. *P*-value** |
| --- | --- | --- | --- |
| *CD177* | CD177 molecule | 3.29 | 1.37E-10 |
| *HP* | haptoglobin | 1.97 | 1.30E-09 |
| *MMP8* | matrix metallopeptidase 8 | 1.91 | 2.94E-09 |
| *ANKRD22* | ankyrin repeat domain 22 | 1.87 | 1.68E-07 |
| *MCEMP1* | mast cell expressed membrane protein 1 | 1.83 | 8.70E-11 |
| *GPR84* | G protein-coupled receptor 84 | 1.79 | 4.83E-11 |
| *MGAM2* | maltase-glucoamylase 2 (putative) | 1.78 | 5.46E-15 |
| *FCGR1A* | Fc fragment of IgG receptor Ia | 1.73 | 5.13E-11 |
| *VNN1* | vanin 1 | 1.67 | 4.40E-12 |
| *FCGR1B* | Fc fragment of IgG receptor Ib | 1.55 | 4.26E-11 |
| *FAM20A* | FAM20A golgi associated secretory pathway pseudokinase | 1.54 | 6.01E-15 |
| *LOC105377499* | uncharacterized LOC105377499 | 1.52 | 5.30E-12 |
| *CASP5* | caspase 5 | 1.50 | 3.42E-09 |
| *CARD17* | caspase recruitment domain family member 17 | 1.50 | 8.10E-08 |
| *ANXA3* | annexin A3 | 1.49 | 8.62E-12 |
| *BMX* | BMX non-receptor tyrosine kinase | 1.47 | 7.38E-10 |
| *MMP9* | matrix metallopeptidase 9 | 1.43 | 2.84E-08 |
| *CD274* | CD274 molecule | 1.39 | 2.44E-07 |
| *BASP1-AS1* | BASP1 antisense RNA 1 | 1.36 | 3.26E-09 |
| *SOCS3* | suppressor of cytokine signaling 3 | 1.34 | 2.34E-10 |
| *LOC105378085* | uncharacterized LOC105378085 | 1.31 | 2.96E-10 |
| *CYP1B1* | cytochrome P450 family 1 subfamily B member 1 | 1.31 | 1.19E-09 |
| *S100A12* | S100 calcium binding protein A12 | 1.29 | 7.98E-14 |
| *SLED1* | proteoglycan 3, pro eosinophil major basic protein 2 pseudogene | 1.28 | 4.70E-09 |
| *CACNA1E* | calcium voltage-gated channel subunit alpha1 E | 1.28 | 1.54E-08 |
| *LOC102723739* | uncharacterized LOC102723739 | 1.26 | 2.23E-05 |
| *MGAM* | maltase-glucoamylase | 1.25 | 3.16E-12 |
| *CLEC5A* | C-type lectin domain containing 5A | 1.24 | 1.09E-08 |
| *METTL7B* | methyltransferase like 7B | 1.24 | 1.78E-07 |
| *KREMEN1* | kringle containing transmembrane protein 1 | 1.23 | 1.95E-11 |
| *ALPL* | alkaline phosphatase, biomineralization associated | 1.23 | 3.70E-11 |
| *SHOC1* | shortage in chiasmata 1 | 1.22 | 2.32E-10 |
| *TLR5* | toll like receptor 5 | 1.21 | 8.42E-13 |
| *ECRP* | ribonuclease A family member 2 pseudogene | 1.21 | 3.59E-08 |
| *NLRC4* | NLR family CARD domain containing 4 | 1.21 | 3.45E-11 |
| *GYG1* | glycogenin 1 | 1.20 | 5.65E-11 |
| *LINC01270* | long intergenic non-protein coding RNA 1270 | 1.20 | 2.42E-10 |
| *MIR4802* | microRNA 4802 | 1.20 | 3.72E-08 |
| *CLEC4D* | C-type lectin domain family 4 member D | 1.19 | 9.48E-09 |
| *SLC26A8* | solute carrier family 26 member 8 | 1.19 | 3.57E-09 |
| *DYSF* | dysferlin | 1.19 | 2.90E-12 |
| *ACSL1* | acyl-CoA synthetase long chain family member 1 | 1.19 | 6.34E-11 |
| *RETN* | resistin | 1.19 | 1.50E-05 |
| *TDRD9* | tudor domain containing 9 | 1.18 | 5.77E-11 |
| *LOC105372578* | uncharacterized LOC105372578 | 1.17 | 1.27E-06 |
| *NAIPP3* | NAIP pseudogene 3 | 1.16 | 1.51E-09 |
| *LILRA6* | leukocyte immunoglobulin like receptor A6 | 1.16 | 9.08E-13 |
| *H2BC18* | H2B clustered histone 18 | 1.14 | 1.62E-08 |
| *LINC02555* | long intergenic non-protein coding RNA 2555 | 1.14 | 1.98E-05 |
| *ARG1* | arginase 1 | 1.13 | 2.29E-06 |
| *LILRA5* | leukocyte immunoglobulin like receptor A5 | 1.13 | 9.35E-12 |
| *CEACAM1* | CEA cell adhesion molecule 1 | 1.12 | 3.17E-07 |
| *KCNJ2* | potassium inwardly rectifying channel subfamily J member 2 | 1.12 | 6.18E-12 |
| *LINC00862* | long intergenic non-protein coding RNA 862 | 1.12 | 4.08E-11 |
| *HK3* | hexokinase 3 | 1.11 | 5.40E-12 |
| *MANSC1* | MANSC domain containing 1 | 1.11 | 2.03E-10 |
| *ADM* | adrenomedullin | 1.10 | 1.22E-11 |
| *C3orf86* | chromosome 3 open reading frame 86 | 1.10 | 1.29E-08 |
| *IL1B* | interleukin 1 beta | 1.10 | 2.09E-11 |
| *GK* | glycerol kinase | 1.10 | 4.23E-09 |
| *IRAK3* | interleukin 1 receptor associated kinase 3 | 1.10 | 2.19E-14 |
| *PSTPIP2* | proline-serine-threonine phosphatase interacting protein 2 | 1.09 | 5.77E-11 |
| *IL1R2* | interleukin 1 receptor type 2 | 1.09 | 7.83E-07 |
| *FAM198B-AS1* | FAM198B antisense RNA 1 | 1.09 | 2.92E-10 |
| *MS4A4A* | membrane spanning 4-domains A4A | 1.08 | 3.12E-08 |
| *KCNJ2-AS1* | KCNJ2 antisense RNA 1 | 1.08 | 1.13E-08 |
| *TNFAIP6* | TNF alpha induced protein 6 | 1.08 | 1.89E-07 |
| *CA4* | carbonic anhydrase 4 | 1.07 | 1.22E-08 |
| *OLFM4* | olfactomedin 4 | 1.07 | 2.22E-04 |
| *LOC101928344* | uncharacterized LOC101928344 | 1.06 | 8.99E-12 |
| *FPR2* | formyl peptide receptor 2 | 1.06 | 8.62E-11 |
| *ZNF438* | zinc finger protein 438 | 1.05 | 1.60E-09 |
| *C1QC* | complement C1q C chain | 1.03 | 6.55E-05 |
| *HORMAD1* | HORMA domain containing 1 | 1.03 | 1.02E-11 |
| *FAM151B* | family with sequence similarity 151 member B | 1.03 | 8.43E-14 |
| *PADI2* | peptidyl arginine deiminase 2 | 1.02 | 1.98E-10 |
| *GALNT14* | polypeptide N-acetylgalactosaminyltransferase 14 | 1.02 | 6.26E-08 |
| *SLPI* | secretory leukocyte peptidase inhibitor | 1.02 | 2.16E-06 |
| *LRG1* | leucine rich alpha-2-glycoprotein 1 | 1.02 | 6.12E-09 |
| *SLC2A3* | solute carrier family 2 member 3 | 1.02 | 3.54E-12 |
| *ADGRG3* | adhesion G protein-coupled receptor G3 | 1.01 | 1.42E-09 |
| *C8orf88* | chromosome 8 open reading frame 88 | 1.01 | 4.23E-11 |
| *TMEM144* | transmembrane protein 144 | 1.00 | 1.03E-09 |
| *PYGL* | glycogen phosphorylase L | 1.00 | 2.02E-11 |
| *PLBD1* | phospholipase B domain containing 1 | 1.00 | 2.30E-16 |
| *SNORA60* | small nucleolar RNA, H/ACA box 60 | -1.00 | 1.04E-09 |
| *GNLY* | granulysin | -1.02 | 1.57E-10 |
| *GZMK* | granzyme K | -1.03 | 7.58E-13 |
| *FGFBP2* | fibroblast growth factor binding protein 2 | -1.04 | 1.65E-08 |
| *KLRF1* | killer cell lectin like receptor F1 | -1.05 | 4.38E-07 |
| *HBZ* | hemoglobin subunit zeta | -1.06 | 3.12E-03 |
| *EIF1AY* | eukaryotic translation initiation factor 1A Y-linked | -1.11 | 6.98E-03 |
| *TXLNGY* | taxilin gamma pseudogene, Y-linked | -1.11 | 1.30E-02 |
| *ALOX15* | arachidonate 15-lipoxygenase | -1.13 | 1.02E-06 |
| *IGHG1* | immunoglobulin heavy constant gamma 1 (G1m marker) | -1.63 | 1.19E-02 |
