# Supplementary material for: Molecular analysis of acute pyelonephritis—excessive innate and attenuated adaptive immunity
Source: Life Sci Alliance. 2024 Dec 20;8(3):e202402926. doi: 10.26508/lsa.202402926 (PMC11662066; doi:10.26508/lsa.202402926)
Supplement: Supplementary file 5 [file LSA-2024-02926_TableS5.docx]

**Table S5**. Top regulated genes in acute samples (adj. *P* < 0.05, FC > 1.5), Cohort I. FC = Fold Change.

| **Symbol** | **Entrez Gene Name** | **FC DMSA+** | **FC DMSA-** | **Type(s)** |
| --- | --- | --- | --- | --- |
| *CD177* | CD177 molecule | 54.2 | 31.5 | other |
| *MCEMP1* | mast cell expressed membrane protein 1 | 11.5 | 7.9 | other |
| *HP* | haptoglobin | 9.9 | 5.2 | peptidase |
| *ANKRD22* | ankyrin repeat domain 22 | 9.3 | 4.4 | transcription regulator |
| *VNN1* | vanin 1 | 8.4 | 4.7 | enzyme |
| *MGAM2* | maltase-glucoamylase 2 (putative) | 8.2 | 4.6 | other |
| *GPR84* | G protein-coupled receptor 84 | 7.8 | 4.2 | GPCR |
| *CASP5* | caspase 5 | 7.3 | 4.8 | peptidase |
| *FAM20A* | golgi associated secretory pathway pseudokinase | 7.0 | 4.5 | other |
| *FCGR1A* | Fc fragment of IgG receptor Ia | 6.8 | 4.6 | transmembrane receptor |
| *SNORA72* | small nucleolar RNA, H/ACA box 72 | -2.8 | -2.1 | other |
| *SNORA60* | small nucleolar RNA, H/ACA box 60 | -2.9 | -3.1 | other |
| *FGFBP2* | fibroblast growth factor binding protein 2 | -3.0 | -1.6 | other |
| *SNORA14B* | small nucleolar RNA, H/ACA box 14B | -3.0 | -2.4 | other |
| *GZMK* | granzyme K | -3.1 | -1.7 | peptidase |
| *IGHG4* | immunoglobulin heavy constant gamma 4 (G4m) | -3.1 | -2.5 | other |
| *LOC105370259* | uncharacterized LOC105370259 | -3.4 | -2.8 | other |
| *ALOX15* | arachidonate 15-lipoxygenase | -3.5 | -2.8 | enzyme |
| *GNLY* | granulysin | -3.7 | -1.9 | other |
| *IGHG3* | immunoglobulin heavy constant gamma 3 (G3m) | -3.9 | -2.8 | other |
| *C8orf88* | chromosome 8 open reading frame 88 | 2.5 |  | other |
| *LOC101926933* | uncharacterized LOC101926933 | 2.4 |  | other |
| *ZC3H3* | zinc finger CCCH-type containing 3 | 2.3 |  | transcription regulator |
| *PRRG4* | proline rich and Gla domain 4 | 2.3 |  | other |
| *SUCNR1* | succinate receptor 1 | 2.2 |  | GPCR |
| *BPI* | bactericidal permeability increasing protein | 2.2 |  | transporter |
| *P2RY14* | purinergic receptor P2Y14 | 2.2 |  | GPCR |
| *FAS-AS1* | FAS antisense RNA 1 | 2.2 |  | other |
| *SLC25A28* | solute carrier family 25 member 28 | 2.2 |  | transporter |
| *LTF* | lactotransferrin | 2.2 |  | peptidase |
| *LOC105375130* | uncharacterized LOC105375130 | -2.3 |  | other |
| *IGLV8-61* | immunoglobulin lambda variable 8-61 | -2.3 |  | other |
| *LOC105373105* | uncharacterized LOC105373105 | -2.3 |  | other |
| *TRDC* | T cell receptor delta constant | -2.4 |  | other |
| *KLRF1* | killer cell lectin like receptor F1 | -2.5 |  | transmembrane receptor |
| *TRDJ4* | T cell receptor delta joining 4 | -2.5 |  | other |
| *S1PR5* | sphingosine-1-phosphate receptor 5 | -2.5 |  | GPCR |
| *ADGRG1* | adhesion G protein-coupled receptor G1 | -2.7 |  | GPCR |
| *IGKV1-5* | immunoglobulin kappa variable 1-5 | -2.7 |  | other |
| *TTTY15* | testis-specific transcript, Y-linked 15 | -10.6 |  | other |
| *PDK4* | pyruvate dehydrogenase kinase 4 |  | 1.9 | kinase |
| *CSF1R* | colony stimulating factor 1 receptor |  | 1.8 | kinase |
| *SERPING1* | serpin family G member 1 |  | 1.8 | other |
| *TUBBP5* | tubulin beta pseudogene 5 |  | 1.8 | other |
| *SASH1* | SAM and SH3 domain containing 1 |  | 1.7 | other |
| *ZNF385A* | zinc finger protein 385A |  | 1.7 | other |
| *C10orf105* | chromosome 10 open reading frame 105 |  | 1.7 | other |
| *PLXND1* | plexin D1 |  | 1.7 | transmembrane receptor |
| *ZFHX3* | zinc finger homeobox 3 |  | 1.7 | transcription regulator |
| *L1TD1* | LINE1 type transposase domain containing 1 |  | 1.7 | other |
| *OLIG2* | oligodendrocyte transcription factor 2 |  | -1.6 | transcription regulator |
| *ZNF506* | zinc finger protein 506 |  | -1.6 | transcription regulator |
| *NPR2* | natriuretic peptide receptor 2 |  | -1.6 | GPCR |
| *GRAMD1C* | GRAM domain containing 1C |  | -1.6 | other |
| *THOC3* | THO complex 3 |  | -1.6 | other |
| *LOC102724851* | uncharacterized LOC102724851 |  | -1.6 | other |
| *DDX11L1/19* | DEAD/H-box helicase 11 like 1 (pseudogene) |  | -1.6 | other |
| *LOC100130298* | hCG1816373-like |  | -1.7 | other |
| *RNU5E-1* | RNA, U5E small nuclear 1 |  | -1.7 | other |
| *RNF182* | ring finger protein 182 |  | -2.2 | enzyme |
