# Supplementary material for: Molecular analysis of acute pyelonephritis—excessive innate and attenuated adaptive immunity
Source: Life Sci Alliance. 2024 Dec 20;8(3):e202402926. doi: 10.26508/lsa.202402926 (PMC11662066; doi:10.26508/lsa.202402926)
Supplement: Supplementary file 8 [file LSA-2024-02926_TableS8.docx]

**Table S8**. Study protocol, Cohort I.

|  | **Visit 1**  **(recruitment)** | **Visit 2** | **Visit 3**  **(endpoint)** |
| --- | --- | --- | --- |
| **Time** | Acute infection | 1 month post infection | 6 months post infection |
| **Informed consent** | X |  |  |
| **Inclusion/exclusion criteria according to clinical pathway** | X |  |  |
| **Urine Culture** | X* |  |  |
| **Urine FEME** | X* |  |  |
| **Blood Culture** | X* |  |  |
| **FBC** | X* |  |  |
| **CRP** | X* |  |  |
| **DNA sample** | X** |  |  |
| **RNA sample** | X** |  | X** |
| **Renal Ultrasound** | X* |  |  |
| **DMSA scan** | X** |  | X |
| **MCU** |  | X** |  |
| **Urine sample** | X** | X** | X** |
| FEME = full and microscopic examination of urine, FBC = full blood count, CRP = C-reactive protein, DMSA = dimercaptosuccinic acid, MCU = micturating cystourethrogram.  * Routine examinations according to clinical pathway  ** Study specific examinations | | | |
