# Supplementary material for: Molecular analysis of acute pyelonephritis—excessive innate and attenuated adaptive immunity
Source: Life Sci Alliance. 2024 Dec 20;8(3):e202402926. doi: 10.26508/lsa.202402926 (PMC11662066; doi:10.26508/lsa.202402926)
Supplement: Supplementary file 9 [file LSA-2024-02926_TableS9.docx]

**Table S9.** Centers participating in the Swedish infant UTI study for Cohort II.

| **City/Hospital** | **Responsible investigator** |
| --- | --- |
| Borås | Laura Raduta |
| Eskilstuna | Caroline Tuetey |
| Falun | Ida Kullberg |
| Gävle | Helena Webering |
| Göteborg | Per Brandström |
| Halmstad | Magnus Lindén |
| Helsingborg | Johan Frank |
| Hudiksvall | Magnus Hellberg |
| Jönköping | Simon Rundquist |
| Kalmar | Petra Hornstra van Echtelt |
| Karlskrona | Anna Westrup |
| Karlstad | Emma Wikholm |
| Kristianstad | Henrik Andersson |
| Luleå | Eva Säve-Söderbergh |
| Lund/Malmö | Therese Rosenblad/  Jorge Sotoca Fernandez |
| Skellefteå | Anna Hedlund |
| Skövde | Eva Olofsson |
| Sollefteå | Britta Björsell |
| Stockholm/Karolinska | Sandra Soeria-Atmadja |
| Stockholm/Sachsska | Karin Rosenborg |
| Sundsvall | Joanna Ganowiak |
| Trollhättan | Marie Keillar |
| Umeå | Staffan Berglund |
| Uppsala | Tryggve Nevéus |
| Visby | Jens Bekkebråten |
| Västerås | Renata Sajevic |
| Växjö | Jonas Sjödin |
| Örebro | Simon Jarrick |
| Östersund | Therese Eneland |
